# Supplementary material for: Novel Driver Strength Index highlights important cancer genes in TCGA PanCanAtlas patients
Source: PeerJ. 2022 Aug 11;10:e13860. doi: 10.7717/peerj.13860 (PMC9375969; doi:10.7717/peerj.13860)
Supplement: Supplemental Information 2 [file peerj-10-13860-s002.zip › COHORTS/genes plots/cohorts/2021_8_16_14_9_distribution_cohorts_females_READ.pdf]

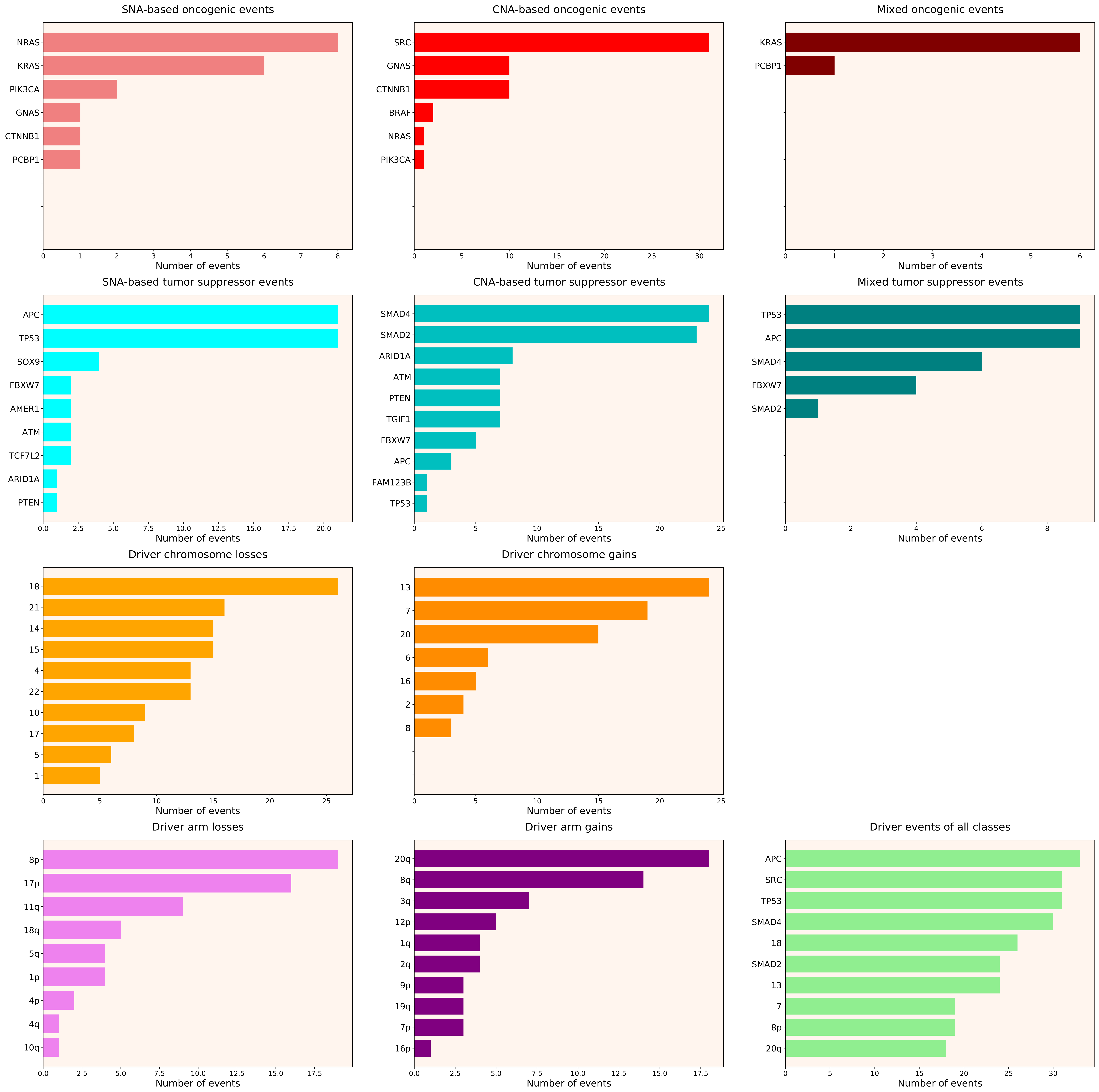
SNA-based oncogenic events
NRAS
KRAS
PIK3CA
GNAS
CTNNB1
PCBP1
0 1 2 3 4 5 6 7 8
Number of events
CNA-based oncogenic events
SRC
GNAS
CTNNB1
BRAF
NRAS
PIK3CA
0 5 10 15 20 25 30
Number of events
Mixed oncogenic events
KRAS
PCBP1
0 1 2 3 4 5 6
Number of events
SNA-based tumor suppressor events
APC
TP53
SOX9
FBXW7
AMER1
ATM
TCF7L2
ARID1A
PTEN
0.0 2.5 5.0 7.5 10.0 12.5 15.0 17.5 20.0
Number of events
CNA-based tumor suppressor events
SMAD4
SMAD2
ARID1A
ATM
PTEN
TGIF1
FBXW7
APC
FAM123B
TP53
0 5 10 15 20 25
Number of events
Mixed tumor suppressor events
TP53
APC
SMAD4
FBXW7
SMAD2
0 2 4 6 8
Number of events
Driver chromosome losses
18
21
14
15
4
22
10
17
5
1
0 5 10 15 20 25
Number of events
Driver chromosome gains
13
7
20
6
16
2
8
0 5 10 15 20 25
Number of events
Driver arm losses
8p
17p
11q
18q
5q
1p
4p
4q
10q
0.0 2.5 5.0 7.5 10.0 12.5 15.0 17.5
Number of events
Driver arm gains
20q
8q
3q
12p
1q
2q
9p
19q
7p
16p
0.0 2.5 5.0 7.5 10.0 12.5 15.0 17.5
Number of events
Driver events of all classes
APC
SRC
TP53
SMAD4
18
SMAD2
13
7
8p
20q
0 5 10 15 20 25 30
Number of events
